# Serial Coronary Artery Calcium Progression and Risk of Major Adverse Cardiovascular Events in an Asian Cohort

**DOI:** 10.3390/jcm15124652

**Published:** 2026-06-16

**Authors:** Jin-Man He, Yu-Chen Wang, Kuan-Cheng Chang

**Affiliations:** 1 Graduate Institute of Biomedical Sciences, China Medical University, Taichung 406040, Taiwan; nurse5907@gmail.com; 2 Division of Cardiology, Department of Internal Medicine, Asia University Hospital, Taichung 41354, Taiwan; 3 Department of Medical Laboratory Science and Biotechnology, Asia University, Taichung 41354, Taiwan; 4 Division of Cardiovascular Medicine, Department of Medicine, China Medical University Hospital, Taichung 40447, Taiwan; 5 School of Medicine, China Medical University, Taichung 406040, Taiwan; 6 Division of Cardiovascular Medicine, Department of Medicine, Taichung Municipal Geriatric Rehabilitation General Hospital-Managed by China Medical University, Taichung 406004, Taiwan

**Keywords:** coronary artery calcium, calcium score progression, major adverse cardiovascular events, statin therapy, residual risk, inverse probability weighting, personalized medicine

## Abstract

**Background/Objectives:** The prognostic value of serial coronary artery calcium (CAC) progression remains uncertain in Asian populations and statin-treated patients. We evaluated the association between CAC progression and subsequent major adverse cardiovascular events (MACE) in a Taiwanese cohort. **Methods:** We retrospectively studied 1791 individuals undergoing two CAC-scoring cardiac CT scans at a tertiary center in Taiwan from 2006 to 2021, excluding those with MACE before the second scan. CAC progression was defined as an annualized Agatston score increase of ≥20 units/year. Time-to-event analyses used landmark Cox models beginning at the second scan, with inverse probability weighting (IPW), balance diagnostics, multivariable Cox regression, and multiple-imputation sensitivity analyses. **Results:** CAC progression occurred in 365 participants (20.4%). Progressors were older and had greater cardiometabolic risk and baseline CAC burden. In a landmark IPW analysis, CAC progression was associated with higher subsequent MACE risk (HR 2.02, 95% CI 1.49–2.74), with a graded association across annualized CAC change categories: HR 1.72 (95% CI 1.17–2.74) for 21–49 units/year and HR 2.86 (95% CI 2.29–3.57) for ≥50 units/year. The association remained consistent in multiple-imputation analysis (HR 1.90, 95% CI 1.36–2.66) and across major clinical subgroups. Discrimination for 10-year MACE was stronger among statin users than non-statin users (AUC 0.774 vs. 0.571), although statin-stratified analyses were exploratory. **Conclusions:** CAC progression was independently associated with subsequent MACE and showed a graded risk relationship. Serial CAC assessment may serve as a useful dynamic marker for refining longitudinal cardiovascular risk stratification, while prospective studies are needed to validate progression-guided management.

## 1. Introduction

Coronary artery calcium (CAC), quantified by the Agatston scoring method, is a well-validated biomarker of subclinical atherosclerosis and a robust predictor of future cardiovascular events [1,2]. CAC scoring has been incorporated into preventive cardiology frameworks and professional society statements as a tool for refining cardiovascular risk stratification in asymptomatic individuals [3,4]. The 2018 American College of Cardiology/American Heart Association (ACC/AHA) cholesterol guidelines endorse CAC scoring for refining risk assessment in individuals at borderline or intermediate 10-year atherosclerotic cardiovascular disease (ASCVD) risk [4]. Accumulating evidence from landmark studies and registries has established a dose-response relationship between baseline CAC burden and incident coronary heart disease (CHD), with high CAC scores conferring risk comparable to established ASCVD [5,6].

While baseline CAC provides a snapshot of cumulative atherosclerotic burden, atherosclerosis is inherently a dynamic process. Serial CAC measurement offers the potential to capture disease activity over time and may complement the broader spectrum of non-invasive cardiovascular imaging modalities used for prevention [7]. The MESA cohort demonstrated that annualized CAC progression independently predicts incident CHD and all-cause mortality beyond baseline CAC scores [8,9]. In the population-based HNR study, CAC progression was associated with incident coronary and cardiovascular events, but provided only limited incremental prognostic value beyond the most recent CAC score and updated risk factor assessment [10]. However, several important questions remain unresolved.

First, the optimal definition of clinically meaningful CAC progression lacks standardization, with different studies employing absolute change, percentage change, square-root transformation, and log-transformed metrics [8,9,10]. Absolute annualized change is clinically intuitive, but it may be influenced by baseline CAC burden because individuals with established calcification have more substrate for subsequent calcium accumulation. Second, the interaction between statin therapy and CAC progression introduces a clinical interpretation paradox: meta-analyses demonstrate that statins may accelerate Agatston score progression while simultaneously reducing cardiovascular events [11,12]. This apparent contradiction reflects the dual nature of the Agatston score, which conflates plaque volume (a marker of disease burden) with calcification density (a marker of plaque stabilization) [13]. Third, the vast majority of serial CAC data originates from Western populations, with limited representation from East Asian cohorts where baseline CAC prevalence and atherosclerosis kinetics may differ [14,15].

Furthermore, whether CAC progression retains prognostic significance in statin-treated patients remains a critical knowledge gap. If statin therapy inherently promotes plaque calcification as a stabilization mechanism, clinicians need to understand whether observed CAC progression in treated patients represents benign plaque remodeling or ongoing pathological disease activity. This distinction may have implications for residual risk assessment and subsequent optimization of preventive therapy.

To address these gaps, we conducted a retrospective cohort study in a Taiwanese population to evaluate: (1) independent predictors of CAC progression; (2) the association between CAC progression and subsequent MACE using a landmark approach beginning at the second CAC scan; and (3) the exploratory prognostic value of CAC progression stratified by statin use. We hypothesized that CAC progression would be independently associated with subsequent MACE after accounting for baseline clinical risk factors and CAC burden, supporting its potential role as a dynamic marker for longitudinal cardiovascular risk assessment.

## 2. Methods

### 2.1. Study Design

This was a retrospective, single-center cohort study conducted at China Medical University Hospital (CMUH), a tertiary medical center in Taichung, Taiwan. We identified all individuals who underwent at least two non-contrast cardiac computed tomography (CT) scans for CAC scoring between January 2006 and December 2021. A total of 2155 individuals were initially identified. After excluding individuals with a second CT scan performed within 7 days of the first scan (*n* = 5), prior coronary stent implantation at CMUH before the baseline scan (*n* = 40), and MACE before the second CAC scan (*n* = 319), the final study cohort comprised 1791 individuals. The study protocol was approved by the Institutional Review Board of CMUH under protocol number CMUH105-REC2-047 (AR-5), and the requirement for informed consent was waived because of the retrospective nature of the study.

### 2.2. Coronary Artery Calcium Scoring

CAC scoring was performed using an electrocardiogram (ECG)-gated non-contrast cardiac CT. Agatston scores were calculated using standard methodology [1]. Baseline CAC was defined as the score from the first qualifying CT scan, and follow-up CAC was defined as the score from the most recent qualifying scan.

### 2.3. Definition of CAC Progression

CAC progression was defined as an annualized change in Agatston score of 20 or more units per year, calculated as: (follow-up CAC score—baseline CAC score)/inter-scan interval in years. This absolute annualized threshold was selected because it is readily interpretable in clinical practice and has been used in prior studies to identify rapid CAC progression [16]. However, because no universally accepted definition of CAC progression exists, we also performed a sensitivity analysis using conventional multivariable Cox regression, multiple-imputation sensitivity analyses, and a lower alternative threshold, defining CAC progression as an absolute annualized CAC increase of ≥15 Agatston units/year. Participants were classified into two groups: a CAC progression group (*n* = 365) and a non-progression group (*n* = 1426).

### 2.4. Baseline Clinical Variables

Demographic and clinical data were extracted from electronic medical records at the time of the baseline CT scan. Variables included age, sex, body mass index (BMI), history of diabetes mellitus, hypertension, hyperlipidemia, and cardiovascular disease. Laboratory parameters included systolic and diastolic blood pressure, high-sensitivity C-reactive protein (hs-CRP), homocysteine, blood urea nitrogen (BUN), creatinine, estimated glomerular filtration rate (GFR), fasting glucose (AC), glycated hemoglobin (HbA1c), total cholesterol, triglycerides, high-density lipoprotein cholesterol (HDL-C), low-density lipoprotein cholesterol (LDL-C), and uric acid. Medication data, including statin use, antihypertensive agents, antithrombotic agents, and antidiabetic medications, were recorded.

### 2.5. Outcome Definition

The primary outcome was the first MACE event occurring after the second CAC scan, defined as a composite of acute myocardial infarction (AMI), stroke, or cardiovascular death. Events were ascertained primarily from CMUH electronic medical records, including discharge diagnoses (ICD-10 coded), cardiac catheterization reports, brain imaging studies, and death records. Stroke was defined as a new ischemic or hemorrhagic stroke confirmed by imaging; transient ischemic attacks were not included. Cardiovascular death was defined as death attributed to ischemic heart disease, heart failure, fatal arrhythmia, or stroke based on death certificates and inpatient records available at CMUH. Because CAC progression can only be determined after the second CAC scan, participants with MACE before the second CAC scan were excluded from the landmark cohort, and person-years were calculated from the date of the second CAC scan until the date of the first subsequent MACE, death, or 31 December 2022, whichever occurred first. Events occurring before the second CAC scan were not counted as post-progression outcomes.

### 2.6. Statistical Analysis

Baseline characteristics were summarized according to CAC progression status and compared using chi-square tests for categorical variables and independent-samples t tests for continuous variables. Predictors of CAC progression were assessed using univariable and multivariable logistic regression models, with results reported as odds ratios (ORs) and 95% confidence intervals (CIs). To address concerns regarding multicollinearity among lipid variables, variance inflation factors (VIFs) were assessed; total cholesterol showed substantial collinearity in the revised model (VIF = 16.04) and was removed. Incidence rates of MACE were calculated per 1000 person-years from the second CAC examination to the first subsequent MACE, death, or 31 December 2022, whichever occurred first. The association between CAC progression and MACE was evaluated using Cox proportional hazards models. Propensity score-based IPW was applied to reduce measured baseline differences between participants with and without CAC progression; propensity scores were estimated using a logistic regression model including sex, fasting glucose, antidiabetic medication use, and baseline CAC score, which were identified from stepwise logistic regression and clinical relevance. Stabilized IPW weights were applied to the complete-case cohort; 1535 participants with complete data for all propensity-score model covariates were included in the weighted analyses, while 256 were excluded because of missing covariate data. Stabilized IPW weights were applied without trimming of extreme weights. Post-weighting balance was assessed using standardized mean differences, and propensity-score and weight distributions were examined. The proportional hazards assumption was formally tested. Because residual covariate imbalance remained after weighting, additional sensitivity analyses were performed using conventional multivariable Cox regression and multiple imputation with 30 imputations. To evaluate the dose-response relationship between annualized CAC change and MACE, participants were categorized according to annualized CAC change. Descriptive event-rate analyses used four categories: 0, 0.1–20, 21–49, and ≥50 Agatston units/year. For time-to-event analyses, non-progression was used as the reference group, and progression categories were evaluated using landmark Cox models beginning at the second CAC scan. Trend tests were performed by modeling ordered CAC-change categories as an ordinal variable. Subgroup analyses were conducted according to sex, age, diabetes, hypertension, cardiovascular disease history, and statin use, with interaction terms tested for each subgroup. The joint association of CAC progression and statin use with MACE was evaluated exploratorily using four groups defined by statin use and CAC progression status, with the non-statin/non-progression group as the reference. Pairwise comparisons were performed using landmark IPW-adjusted Cox models. Receiver operating characteristic curve analysis was used to assess the ability of CAC progression to predict 10-year MACE according to statin use. All analyses were performed using SAS version 9.4, and a two-tailed *p* value < 0.05 was considered statistically significant.

### 2.7. Use of Generative Artificial Intelligence Tools

OpenAI ChatGPT (GPT-5.5 Thinking model, OpenAI, San Francisco, CA, USA; accessed on 15 June 2026) was used only for English language editing, grammar correction, and improvement of readability during manuscript preparation. The tool was not used for study design, data collection, data analysis, statistical programming, interpretation of results, figure or table generation, or formulation of scientific conclusions. All AI-assisted language revisions were reviewed, edited, and approved by the authors, who take full responsibility for the content of the manuscript.

## 3. Results

### 3.1. Study Cohort and Group Categorization

A total of 2155 individuals who underwent serial cardiac computed tomography between 2006 and 2021 were initially identified. After exclusions for a second scan within 7 days, prior coronary stent implantation, and MACE before the second CAC scan, the final cohort included 1791 individuals. Participants were categorized according to annualized CAC score change: 365 individuals were classified into the CAC progression group, and 1426 were assigned to the non-progression group. The median inter-scan interval was 3.79 years (IQR 2.44–5.50), with a mean of 4.29 years (SD 2.48; range 0.02–15.16).

### 3.2. Baseline Characteristics

Baseline demographic and clinical characteristics stratified by CAC progression are presented in Table 1. Compared with the non-progression group, individuals with CAC progression were older (58.1 ± 9.42 vs. 52.8 ± 9.22 years, *p* < 0.0001) and had a higher baseline cardiovascular risk profile. The progression group had a higher BMI (26.8 ± 4.09 vs. 25.2 ± 3.51 kg/m^2^, *p* < 0.0001), greater prevalence of diabetes mellitus (17.3% vs. 7.22%, *p* < 0.0001), hypertension (33.4% vs. 18.2%, *p* < 0.0001), hyperlipidemia (29.3% vs. 19.9%, *p* = 0.0001), and cardiovascular disease (18.9% vs. 11.0%, *p* < 0.0001), as well as higher baseline CAC scores (342.2 ± 457.0 vs. 42.1 ± 217.9, *p* < 0.0001). Among laboratory parameters, participants with CAC progression had lower HDL-C levels; higher triglyceride, fasting glucose, HbA1c, BUN, creatinine, and uric acid levels; and lower GFR, indicating a less favorable metabolic and renal profile, whereas LDL-C levels did not differ significantly between groups.

The distribution of baseline CAC categories also differed markedly between groups (Table 2, *p* < 0.0001). In the progression group, 66.6% had baseline CAC > 100, 31.2% had CAC between 0.1 and 100, and only 2.19% had CAC = 0. In contrast, the non-progression group was predominantly composed of individuals with CAC = 0 (61.6%), with 31.3% having CAC between 0.1 and 100 and 7.08% having CAC > 100. These data indicate that CAC progression was observed predominantly among individuals with established baseline calcification.

### 3.3. Factors Associated with CAC Progression in Logistic Regression Analyses

Univariable and multivariable logistic regression analyses identifying predictors of CAC progression are presented in Table 3. In univariable analysis, CAC progression was associated with older age, male sex, higher BMI, diabetes, hypertension, hyperlipidemia, cardiovascular disease, higher systolic blood pressure, homocysteine, BUN, fasting glucose, triglycerides, uric acid, lower GFR, lower HDL-C, and higher baseline CAC score.

After multivariable adjustment in the final model, in which total cholesterol was excluded because of collinearity, male sex remained strongly associated with CAC progression (adjusted OR 8.27; 95% CI 2.01–34.1; *p* = 0.003). Fasting glucose was independently associated with progression (adjusted OR 1.02; 95% CI 1.01–1.03; *p* = 0.003), and baseline CAC score remained the strongest imaging predictor (adjusted OR 1.14 per 10-unit increase; 95% CI 1.09–1.18; *p* < 0.0001). Hypertension (adjusted OR 2.36; 95% CI 0.94–5.91; *p* = 0.068) and LDL-C (adjusted OR 1.11 per 10 mg/dL; 95% CI 0.99–1.24; *p* = 0.078) showed borderline associations, whereas HDL-C was not independently associated with CAC progression (adjusted OR 1.20 per 10 mg/dL; 95% CI 0.85–1.69; *p* = 0.305).

### 3.4. CAC Progression and Risk of MACE

Of the 1791 participants in the final cohort, 1535 had complete data on all covariates required for the propensity score model and were included in the IPW-adjusted Cox analyses. The median follow-up duration from the second CAC scan was 5.12 years (IQR 3.04–7.93). To evaluate the potential impact of complete-case inclusion on the weighted analyses, baseline characteristics were compared between participants included in and excluded from the IPW-adjusted Cox models (Table S1). Several differences were observed between the included and excluded participants, including diabetes mellitus, hyperlipidemia, BUN, total cholesterol, LDL-C, uric acid, calcium-channel blocker use, renin-angiotensin system inhibitor use, antidiabetic medication use, and statin use. However, the proportion of MACE did not differ significantly between the two groups (10.8% vs. 7.81%; *p* = 0.145). These findings suggest that exclusion due to missing propensity-score model covariates was unlikely to fully explain the observed association between CAC progression and MACE, although residual selection bias cannot be excluded.

**Figure 1:**
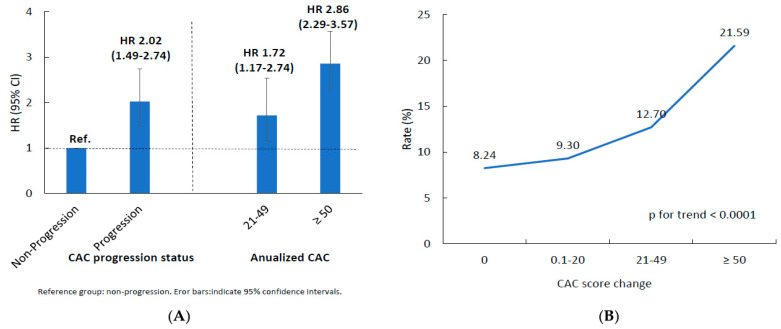
Association Between CAC Progression and Risk of MACE. (**A**) Landmark Cox analysis beginning at the second CAC scan showing hazard ratios for subsequent MACE according to CAC progression status and annualized CAC score change categories. CAC progression was defined as an annualized CAC increase ≥20 units/year. The HR was 2.02 (95% CI 1.49–2.74) for overall progression, 1.72 (95% CI 1.17–2.74) for 21–49 units/year, and 2.86 (95% CI 2.29–3.57) for ≥50 units/year compared with non-progression. Error bars indicate 95% confidence intervals. (**B**) Observed MACE rates across annualized CAC score change categories. MACE rates increased progressively with greater CAC change (*p* for trend < 0.0001).

In the landmark analysis, 124 MACE occurred in the non-progression group and 62 events occurred in the progression group, corresponding to incidence rates of 15.19 and 31.04 per 1000 person-years, respectively. As shown in Figure 1A, CAC progression was associated with a higher risk of subsequent MACE compared with non-progression in the landmark IPW-adjusted Cox analysis (HR 2.02; 95% CI 1.49–2.74). When annualized CAC change was further categorized, the HRs were 1.72 (95% CI 1.17–2.74) for 21–49 units/year and 2.86 (95% CI 2.29–3.57) for ≥50 units/year, compared with non-progression. No violation of the proportional hazards assumption was detected (*p* = 0.337). Further descriptive analysis demonstrated a stepwise increase in the observed MACE rate across categories of annualized CAC change. As shown in Figure 1B, MACE rates increased from 8.24% among participants with no CAC increase to 9.30%, 12.70%, and 21.59% among those with annualized CAC increases of 0.1–20, 21–49, and ≥50 units/year, respectively (*p* for trend < 0.0001). These findings support a graded relationship between the magnitude of CAC progression and observed cardiovascular event burden. Landmark cumulative-incidence curves beginning at the second CAC scan showed clear separation between participants with and without CAC progression (log-rank test, *p* < 0.0001), consistent with the landmark Cox analysis (Figure S1).

Post-weighting diagnostics were added to evaluate covariate balance after IPW. The SMDs before and after IPTW were 0.534 and 0.735 for sex, 0.454 and 0.035 for fasting glucose, 0.206 and 0.024 for antidiabetic medication use, and 0.838 and 0.344 for baseline CAC score, respectively. The median propensity score was 0.14 (IQR 0.06–0.16) in non-progressors and 0.27 (IQR 0.19–0.52) in progressors; the corresponding median weights were 1.17 (IQR 1.06–1.20) and 2.53 (IQR 1.33–4.75), respectively. These diagnostics indicate partial but incomplete balance, particularly for sex and baseline CAC score. Therefore, IPW-adjusted estimates were interpreted cautiously, and additional sensitivity analyses were performed. CAC progression remained associated with MACE in a conventional multivariable Cox model (HR 1.85; 95% CI, 1.29–2.64; Table S2), and a multiple-imputation analysis with 30 imputations yielded a consistent estimate (HR 1.90; 95% CI, 1.36–2.66; Table S3). In an additional sensitivity analysis using an alternative CAC progression threshold of ≥15 Agatston units/year, the direction of association remained consistent, although the IPW-adjusted estimate was markedly inflated and showed limited separation between progression categories (Table S4). This analysis was therefore interpreted cautiously and considered supportive only of the direction of association. Taken together, these sensitivity analyses support an association between CAC progression and subsequent MACE, while indicating that the magnitude of the IPW-adjusted estimates may be influenced by residual imbalance and limited common support.

### 3.5. Joint Effects of CAC Progression and Statin Use

Among the 1791 participants, 144 (8.0%) were receiving statin therapy. Statin users were older, had a higher prevalence of diabetes, hypertension, hyperlipidemia, cardiovascular disease, medication use, and higher baseline CAC scores compared with non-statin users (Table 4). Medication use according to CAC progression status is summarized in Table S5. Compared with non-progressors, participants with CAC progression were more frequently treated with cardiac drugs, diuretics, calcium-channel blockers, renin-angiotensin system inhibitors, antithrombotic agents, antidiabetic medications, and statins, reflecting a higher baseline cardiovascular risk profile and greater treatment burden. In the medication-based regression analysis, statin use was associated with CAC progression in univariable analysis but was attenuated and no longer statistically significant after multivariable adjustment (Table S6), consistent with confounding by indication.

**Figure 2:**
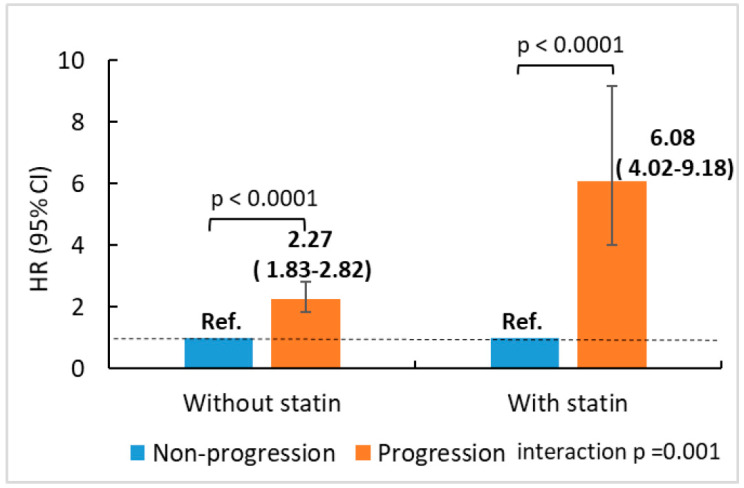
Adjusted Hazard Ratios for MACE According to Joint Stratification by CAC Progression and Statin Use. Exploratory statin-stratified landmark analysis of hazard ratios for MACE according to CAC progression status. The horizontal dashed line indicates an HR of 1.0, representing no difference in MACE risk compared with the corresponding non-progression reference group within each statin stratum. Within each statin stratum, the non-progression group served as the reference. CAC progression was associated with higher MACE risk among participants without statin therapy (HR 2.27; 95% CI 1.83–2.82) and among participants with statin therapy (HR 6.08; 95% CI 4.02–9.18); interaction *p* = 0.001.

The joint effects of CAC progression and statin use on MACE risk are presented as exploratory analyses. As shown in Figure 2, in the statin-stratified landmark analysis, CAC progression was associated with higher MACE risk both among participants without statin therapy (HR 2.27; 95% CI 1.83–2.82) and among participants with statin therapy (HR 6.08; 95% CI 4.02–9.18), using the corresponding non-progression subgroup as reference. Additional four-group analyses are shown in Figure S2. Using non-progression without statin therapy as the reference, the HRs were 2.27 (95% CI 1.82–2.82) for progression without statin therapy, 0.59 (95% CI 0.58–0.61) for non-progression with statin therapy, and 2.95 (95% CI 1.95–4.45) for progression with statin therapy. The CAC progression × statin interaction was statistically significant in the landmark analysis (interaction *p* = 0.001). Given the non-randomized and binary ascertainment of statin exposure, these statin-stratified findings should be considered hypothesis-generating.

### 3.6. Subgroup Analyses

Subgroup analyses examining the association between CAC progression and MACE risk are presented in Table 5. CAC progression was consistently associated with higher incidence rates across all examined clinical subgroups. The relative association was particularly large in women (HR 7.11; 95% CI 4.09–12.3), whereas the association in men was more modest but remained significant (HR 2.34; 95% CI 1.91–2.87), with a significant interaction by sex (interaction *p* < 0.0001). For age, diabetes, hypertension, and cardiovascular disease history, the direction of association was consistent and interaction testing was not statistically significant. These subgroup findings should be interpreted as exploratory assessments of consistency rather than definitive evidence of subgroup-specific risk effects, because multiple interaction tests were performed and some strata had relatively few events.

Importantly, absolute incidence rates remained higher among CAC progressors in clinically relevant subgroups. For example, women with CAC progression had an incidence rate of 47.20 versus 17.23 per 1000 person-years in non-progressors, and patients with diabetes had rates of 45.83 versus 14.91 per 1000 person-years, respectively.

### 3.7. ROC Curve Analysis Stratified by Statin Use

**Figure 3:**
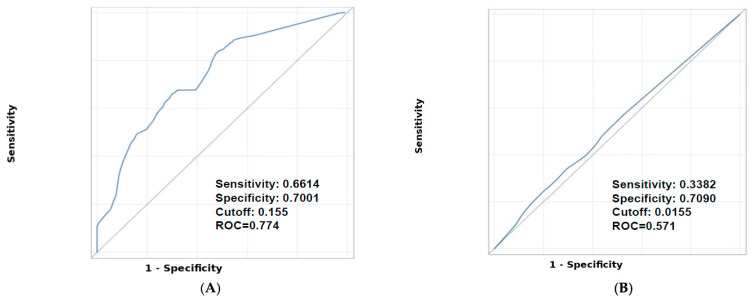
Receiver Operating Characteristic Curve Analysis of CAC Progression for Predicting 10-Year MACE According to Statin Use. In each panel, the colored curve represents the ROC curve for CAC progression, whereas the diagonal gray line represents the no-discrimination reference line (AUC = 0.5). (**A**) ROC curve for CAC progression predicting 10-year MACE among statin users, with an AUC of 0.774, sensitivity of 66.1%, specificity of 70.0%, and optimal cutoff of 0.155. (**B**) ROC curve for CAC progression predicting 10-year MACE among non-statin users, with an AUC of 0.571, sensitivity of 33.8%, specificity of 70.9%, and optimal cutoff of 0.0155.

ROC curve analyses evaluating the performance of CAC progression for predicting 10-year MACE according to statin use are presented in Figure 3. Among statin users (Figure 3A), CAC progression demonstrated moderate discriminative ability (AUC = 0.774; sensitivity 66.1%; specificity 70.0%; optimal cutoff 0.155). Among non-statin users (Figure 3B), CAC progression showed weaker discrimination (AUC = 0.571; sensitivity 33.8%; specificity 70.9%; optimal cutoff 0.0155). These findings suggest that CAC progression alone should not be used as a stand-alone prediction tool, but its stronger performance among statin-treated participants supports the hypothesis that ongoing progression may identify a selected residual-risk phenotype that warrants clinical reassessment.

## 4. Discussion

In this retrospective Taiwanese serial-CAC cohort, CAC progression was associated with a higher risk of subsequent MACE. Using the second CAC scan as the time origin, the primary IPW-adjusted analysis showed an approximately two-fold increase in MACE risk among progressors (HR 2.02; 95% CI 1.49–2.74). This association was directionally consistent in conventional multivariable Cox and multiple-imputation sensitivity analyses. While causal inference remains limited, these findings support the clinical relevance of serial CAC change for refining longitudinal cardiovascular risk assessment.

### 4.1. CAC Progression as an Independent Predictor of MACE

Our results are consistent with prior landmark studies demonstrating the prognostic value of serial CAC assessment. The MESA cohort established that CAC progression predicts incident CHD and all-cause mortality beyond baseline CAC [8,9], and the Heinz Nixdorf Recall study confirmed that CAC progression is associated with incident coronary and cardiovascular events, particularly among individuals with baseline CAC > 0 [10]. Our study extends these observations to a Taiwanese population, but also highlights the methodological challenges of evaluating CAC progression in a highly selected serial-imaging cohort with substantial baseline differences between progressors and non-progressors.

The graded relationship between larger annualized CAC increases and higher observed MACE rates supports the clinical relevance of serial calcium accumulation. Nevertheless, absolute annualized change is influenced by baseline CAC burden: individuals with established calcification are inherently more likely to experience larger absolute increases than those with CAC = 0. For this reason, CAC progression should be interpreted in the context of baseline CAC category, inter-scan interval, and overall cardiovascular risk rather than as an isolated binary marker.

The subgroup findings should also be interpreted cautiously. Although CAC progression was consistently associated with higher MACE rates across clinical subgroups, multiple interaction testing increases the possibility of chance findings. The sex interaction observed in the analysis is clinically interesting but requires external validation, particularly because the number of women with CAC progression was small.

The landmark IPW-adjusted estimate was clinically interpretable and directionally consistent with the conventional Cox and multiple-imputation analyses. However, incomplete post-weighting balance, particularly for sex and baseline CAC burden, suggests that the magnitude of association should be interpreted cautiously as an adjusted observational estimate rather than a causal effect size.

### 4.2. Independent Predictors of CAC Progression

The multivariable model identified male sex, fasting glucose, and baseline CAC score as independent predictors of CAC progression, while hypertension and LDL-C showed borderline associations. These findings are clinically plausible and align with known drivers of atherosclerotic progression. Although HDL-C differed between groups at baseline, it was not independently associated with CAC progression in the final multivariable model, suggesting that the baseline difference likely reflected the broader cardiometabolic risk profile rather than an independent association.

The finding that baseline CAC score independently predicts future progression is consistent with the concept of “atherosclerotic momentum” -patients with established calcification have a higher substrate for continued plaque development. This aligns with our distributional analysis showing that 66.6% of progressors had baseline CAC > 100, while only 2.19% had CAC = 0. Together with prior evidence from the HNR study showing that repeat CAC assessment provides the greatest risk readjustment in individuals with pre-existing CAC, these findings reinforce the clinical principle that serial monitoring is most informative in patients with established calcification [10].

### 4.3. The Statin Paradox and Residual Risk

The statin-stratified analyses are clinically interesting but should remain exploratory. Only 144 participants (8.0%) were receiving statins, and statin exposure was recorded only as a binary variable. Information on statin intensity, duration, adherence, timing relative to CAC scans, achieved LDL-C levels, and treatment changes during follow-up was unavailable. These limitations substantially restrict the mechanistic interpretation of the observed statin-related patterns, even though the interaction term was statistically significant.

The apparent statin-CAC paradox remains important for clinical interpretation. Statins may increase calcification density and Agatston score while reducing plaque vulnerability and cardiovascular events [11,12,17,18,19]. Therefore, CAC progression during statin therapy should not automatically be interpreted as treatment failure. Conversely, progression in a statin-treated patient may still identify a higher-risk phenotype requiring reassessment of residual risk, including LDL-C, lipoprotein(a), inflammation, blood pressure, diabetes, and adherence to preventive therapy. Whether such reassessment should be guided by serial CAC requires prospective validation.

The higher MACE risk observed among statin-treated participants with CAC progression should not be interpreted as evidence that statin therapy is harmful. Statin users in this cohort had substantially higher baseline cardiovascular risk, greater medication burden, and greater atherosclerotic burden than non-statin users, reflecting confounding by indication. Therefore, this study was not designed to determine whether statin therapy modifies the biological effect of CAC progression on MACE. Rather, the statin-stratified findings support a cautious hypothesis: when CAC progression is observed despite statin therapy, it may identify a subgroup with persistent residual risk that deserves reassessment of lipid control, inflammation, blood pressure, diabetes, adherence, and other modifiable risk factors.

### 4.4. Implications for Personalized Cardiovascular Risk Management

Our findings support the potential role of serial CAC assessment as a tool for dynamic risk monitoring in selected patients, particularly those with established baseline calcification. A recently proposed CAC staging framework classifies patients according to CAC burden and links higher stages to more intensive preventive interventions [20]. Within such a framework, CAC progression may be considered a form of dynamic risk reclassification or potential stage migration, but its clinical use should be integrated with baseline CAC, traditional risk factors, and treatment status.

Although CAC progression alone demonstrated only limited-to-moderate discrimination, these findings should not diminish its clinical relevance when serial imaging is already available. Serial CAC change provides a dynamic measure of atherosclerotic activity that complements baseline CAC burden and traditional risk factors. Evidence of CAC progression may help identify individuals with ongoing disease activity and prompt clinicians to reassess the intensity of preventive strategies, including lipid lowering, blood pressure control, glycemic management, and other residual-risk modifiers. In this context, CAC progression should be viewed not as an isolated prediction tool, but as a clinically meaningful imaging marker that can refine longitudinal cardiovascular risk assessment.

The 2025 American Heart Association scientific statement supporting opportunistic CAC detection from non-gated chest CTs may substantially expand the population in whom CAC is recognized [21], potentially facilitating broader risk reassessment and, in selected patients, consideration of longitudinal CAC monitoring. Our finding that CAC progression consistently predicts MACE across subgroups supports the clinical utility of incorporating serial CAC data when follow-up imaging is available.

### 4.5. Comparison with Existing Literature

Our study complements and extends the existing evidence base in several ways. While the MESA cohort and Heinz Nixdorf Recall study have established the prognostic value of CAC progression in predominantly Western populations [8,9,10], our study provides validation in an East Asian cohort. The Asian meta-analysis by Chen et al. confirmed a 5-year warranty period for CAC = 0 in Asian populations and demonstrated sex-specific progression patterns [14], both of which are consistent with our distributional data. The use of propensity score-based inverse probability weighting in our analysis allowed us to reduce measured baseline imbalances between progression and non-progression groups; however, residual and unmeasured confounding cannot be excluded, and our findings should be interpreted as associations rather than causal effects.

The statin-CAC interaction has been examined in prior observational studies [18,19] and meta-analyses [11,12], but few have employed joint stratification with IPW-adjusted Cox models to directly compare MACE risk between statin-using progressors and non-progressors. Our study design thus provides a useful complement to existing literature on this critical clinical question.

## 5. Limitations

Several limitations should be acknowledged. First, this was a retrospective, single-center study of individuals undergoing repeated CAC scans at a tertiary medical center, introducing referral and surveillance bias and limiting generalizability to community-based populations. Second, outcomes were ascertained primarily through CMUH records; MACE treated exclusively at outside hospitals may have been missed, and linkage to nationwide claims or registry data would improve outcome ascertainment. Third, CAC progression was defined using the Agatston score, which conflates plaque volume and density; we could not distinguish disease-driven calcium accumulation from potentially stabilizing increases in calcification density. Fourth, the ≥20 Agatston units/year threshold is clinically interpretable but not universally accepted, and absolute annualized change is influenced by baseline CAC burden and inter-scan interval. Fifth, although IPW was applied, post-weighting SMDs showed that complete covariate balance was not achieved, particularly for sex and baseline CAC, indicating residual imbalance and possible limited common support. Sixth, 256 participants were excluded from complete-case IPW analyses because of missing covariates; multiple imputation yielded consistent results, but missing-not-at-random mechanisms cannot be excluded. Seventh, statin exposure was recorded only as present or absent, without information on intensity, duration, adherence, timing relative to CAC scans, achieved LDL-C levels, or medication changes. Eighth, subgroup and interaction analyses involved multiple comparisons and should be considered exploratory. Finally, no randomized trial has demonstrated that CAC progression-guided treatment intensification improves hard cardiovascular outcomes; therefore, our observational findings cannot establish causality.

## 6. Conclusions

In this Taiwanese cohort undergoing serial cardiac CT, CAC progression was associated with an increased risk of subsequent MACE and showed a graded relationship with the magnitude of CAC change. These findings support CAC progression as a clinically meaningful dynamic marker for longitudinal cardiovascular risk assessment, particularly in patients with established calcification or persistent risk despite preventive therapy. Although prospective validation is needed, CAC progression should not be dismissed as merely a benign imaging phenomenon. Future studies with detailed medication exposure, plaque characterization, and comprehensive outcome linkage are warranted to determine whether progression-guided risk modification can improve clinical outcomes.

## Figures and Tables

**Table 1:** Baseline Demographic and Clinical Characteristics According to Coronary Artery Calcium Score Progression.

Variable	Progression (*n* = 365)	Non-Progression (*n* = 1426)	*p*-Value
Age, years, mean (SD)	58.1 (9.42)	52.8 (9.22)	<0.0001
Male sex, *n* (%)	332 (91.0)	1007 (70.6)	<0.0001
BMI, kg/m^2^, mean (SD)	26.8 (4.09)	25.2 (3.51)	<0.0001
Diabetes mellitus, *n* (%)	63 (17.3)	103 (7.22)	<0.0001
Hypertension, *n* (%)	122 (33.4)	260 (18.2)	<0.0001
Hyperlipidemia, *n* (%)	107 (29.3)	284 (19.9)	0.0001
Cardiovascular disease, *n* (%)	69 (18.9)	157 (11.0)	<0.0001
SBP, mmHg, mean (SD)	133.7 (18.5)	126.0 (17.8)	<0.0001
DBP, mmHg, mean (SD)	81.5 (11.6)	78.0 (12.1)	0.0004
hs-CRP, mg/L, mean (SD)	0.18 (0.23)	0.27 (0.76)	0.004
Homocysteine, μmol/L, mean (SD)	10.9 (3.32)	9.96 (3.27)	0.0001
BUN, mg/dL, mean (SD)	12.8 (5.14)	11.8 (4.08)	0.001
Creatinine, mg/dL, mean (SD)	0.97 (0.50)	0.88 (0.39)	0.002
GFR, mL/min/1.73 m^2^, mean (SD)	85.9 (14.5)	92.3 (14.3)	<0.0001
Fasting glucose (AC), mg/dL, mean (SD)	112.1 (29.5)	99.8 (24.5)	<0.0001
HbA1c, %, mean (SD)	6.39 (1.11)	5.90 (0.78)	<0.0001
Total cholesterol, mg/dL, mean (SD)	200.9 (42.6)	203.0 (38.5)	0.433
Triglycerides, mg/dL, mean (SD)	169.5 (106.9)	149.9 (120.6)	0.005
HDL-C, mg/dL, mean (SD)	41.6 (10.1)	45.9 (12.9)	<0.0001
LDL-C, mg/dL, mean (SD)	124.7 (37.9)	124.6 (33.4)	0.963
Uric acid, mg/dL, mean (SD)	6.64 (1.59)	6.21 (1.44)	<0.0001
Baseline CAC score, mean (SD)	342.2 (457.0)	42.1 (217.9)	<0.0001

Abbreviations: BMI, body mass index; SBP, systolic blood pressure; DBP, diastolic blood pressure; hs-CRP, high-sensitivity C-reactive protein; BUN, blood urea nitrogen; GFR, glomerular filtration rate; HbA1c, glycated hemoglobin; HDL-C, high-density lipoprotein cholesterol; LDL-C, low-density lipoprotein cholesterol; CAC, coronary artery calcium.

**Table 2:** Distribution of Baseline CAC Categories in the Progression and Non-Progression Groups.

Baseline CAC Category	Progression (*n* = 365) *n* (%)	Non-Progression (*n* = 1426) *n* (%)	*p*-Value
CAC > 100	243 (66.6)	101 (7.08)	<0.0001
0.1 ≤ CAC ≤ 100	114 (31.2)	446 (31.3)	
CAC = 0	9 (2.19)	879 (61.6)	

Abbreviations: CAC, coronary artery calcium.

**Table 3:** Independent Predictors of Coronary Artery Calcium Score Progression Using Univariable and Multivariable Logistic Regression.

Variable	Crude OR	95% CI	*p*-Value	Adjusted OR	95% CI	*p*-Value
Age	1.06	(1.05–1.08)	<0.0001	1.04	(0.99–1.09)	0.104
Male vs. female	4.19	(2.88–6.09)	<0.0001	8.27	(2.01–34.1)	0.003
BMI	1.12	(1.07–1.17)	<0.0001	1.00	(0.89–1.12)	0.996
Diabetes	2.68	(1.91–3.76)	<0.0001	2.44	(0.77–7.71)	0.128
Hypertension	2.25	(1.74–2.91)	<0.0001	2.36	(0.94–5.91)	0.068
Hyperlipidemia	1.67	(1.29–2.16)	0.0001	0.57	(0.21–1.55)	0.273
Cardiovascular disease	1.88	(1.38–2.57)	<0.0001	1.50	(0.45–4.99)	0.513
SBP	1.02	(1.01–1.03)	0.0006	1.01	(0.99–1.03)	0.249
hs-CRP	0.66	(0.40–1.08)	0.101	0.29	(0.07–2.04)	0.214
Homocysteine	1.09	(1.04–1.13)	0.0002	1.03	(0.91–1.16)	0.684
BUN	1.05	(1.02–1.08)	0.0005	1.02	(0.92–1.13)	0.709
GFR	0.97	(0.96–0.98)	<0.0001	1.00	(0.98–1.03)	0.787
Fasting glucose (AC)	1.02	(1.01–1.02)	<0.0001	1.02	(1.01–1.03)	0.003
Triglycerides	1.00	(1.00–1.00)	0.011	1.00	(0.99–1.01)	0.112
HDL-C (per 10 units)	0.73	(0.65–0.82)	<0.0001	1.20	(0.85–1.69)	0.305
LDL-C (per 10 units)	1.00	(0.97–1.04)	0.960	1.11	(0.99–1.24)	0.078
Uric acid	1.22	(1.12–1.33)	<0.0001	1.19	(0.91–1.54)	0.206
Baseline CAC score (per 10 units)	1.05	(1.04–1.05)	<0.0001	1.14	(1.09–1.18)	<0.0001

Abbreviations: OR, odds ratio; CI, confidence interval; BMI, body mass index; SBP, systolic blood pressure; hs-CRP, high-sensitivity C-reactive protein; BUN, blood urea nitrogen; GFR, glomerular filtration rate; HDL-C, high-density lipoprotein cholesterol; LDL-C, low-density lipoprotein cholesterol; CAC, coronary artery calcium. Note: For clinical interpretability, SBP, HDL-C, LDL-C, and baseline CAC score were modeled per 10-unit increase, whereas other continuous variables were modeled per 1-unit increase.

**Table 4:** Baseline Characteristics of Patients With and Without Statin Therapy.

Variable	Non-Statin (*n* = 1647)	Statin (*n* = 144)	*p*-Value
Age, years, mean (SD)	53.5 (9.46)	58.3 (8.79)	<0.0001
Male sex, *n* (%)	1233 (74.9)	106 (73.6)	0.740
BMI, kg/m^2^, mean (SD)	25.5 (3.72)	25.9 (3.32)	0.396
Diabetes mellitus, *n* (%)	117 (7.10)	49 (34.0)	<0.0001
Hypertension, *n* (%)	286 (17.4)	96 (66.7)	<0.0001
Hyperlipidemia, *n* (%)	259 (15.7)	132 (91.7)	<0.0001
Cardiovascular disease, *n* (%)	156 (9.47)	70 (48.6)	<0.0001
SBP, mmHg, mean (SD)	127.3 (18.1)	130.5 (18.5)	0.122
DBP, mmHg, mean (SD)	78.8 (12.0)	77.9 (12.3)	0.503
hs-CRP, mg/L, mean (SD)	0.25 (0.70)	0.27 (0.65)	0.836
Homocysteine, μmol/L, mean (SD)	10.1 (3.33)	10.3 (2.78)	0.750
BUN, mg/dL, mean (SD)	11.9 (4.32)	12.6 (4.32)	0.121
Creatinine, mg/dL, mean (SD)	0.90 (0.43)	0.88 (0.20)	0.372
GFR, mL/min/1.73 m^2^, mean (SD)	91.2 (14.7)	88.5 (13.5)	0.032
Fasting glucose (AC), mg/dL, mean (SD)	101.4 (25.7)	111.6 (27.9)	<0.0001
HbA1c, %, mean (SD)	5.96 (0.86)	6.49 (0.97)	<0.0001
Total cholesterol, mg/dL, mean (SD)	204.4 (28.4)	184.3 (43.8)	<0.0001
Triglycerides, mg/dL, mean (SD)	138.6 (79.8)	155.5 (121.2)	0.024
HDL-C, mg/dL, mean (SD)	44.9 (12.4)	45.5 (12.6)	0.621
LDL-C, mg/dL, mean (SD)	126.4 (33.5)	107.6 (37.4)	<0.0001
Uric acid, mg/dL, mean (SD)	6.30 (1.48)	6.31 (1.52)	0.936
Baseline CAC score, mean (SD)	95.4 (296.3)	193.4 (410.0)	0.006
Antihypertensive, *n* (%)	15 (0.91)	1 (0.69)	1.000
Cardiac drug, *n* (%)	73 (4.43)	46 (31.9)	<0.0001
Diuretics, *n* (%)	24 (1.46)	18 (12.5)	<0.0001
Beta-blocker, *n* (%)	31 (1.88)	11 (7.64)	0.0003
CCB, *n* (%)	85 (5.16)	53 (36.8)	<0.0001
RAS inhibitor, *n* (%)	57 (3.46)	42 (29.2)	<0.0001
Antithrombotic, *n* (%)	81 (4.92)	78 (54.2)	<0.0001
Antidiabetic, *n* (%)	33 (2.00)	21 (14.6)	<0.0001

Abbreviations: BMI, body mass index; BUN, blood urea nitrogen; CAC, coronary artery calcium; CCB, calcium channel blocker; GFR, glomerular filtration rate; HDL-C, high-density lipoprotein cholesterol; hs-CRP, high-sensitivity C-reactive protein; LDL-C, low-density lipoprotein cholesterol; RAS, renin-angiotensin system; SBP, systolic blood pressure; SD, standard deviation.

**Table 5:** Incidence Rates and IPW-Adjusted Hazard Ratios for MACE According to CAC Progression Across Clinical Subgroups.

Subgroup	Non-Prog *n*	Events	IR	Prog *n*	Events	IR	Adj HR (95% CI)	*p*-Value	Interaction *p*
Sex									<0.0001
Women	419	37	17.23	33	8	47.20	7.11 (4.09–12.3)	<0.0001	
Men	1007	87	14.46	332	54	29.54	2.34 (1.91–2.87)	<0.0001	
Age									0.828
<65 years	1280	100	13.56	283	39	24.85	2.12 (1.65–2.72)	<0.0001	
≥65 years	146	24	30.39	82	23	53.70	2.24 (1.66–3.01)	<0.0001	
Diabetes									0.136
No	1323	115	15.21	302	47	28.14	2.22 (1.76–2.81)	<0.0001	
Yes	103	9	14.91	63	15	45.83	3.11 (2.22–4.35)	<0.0001	
Hypertension									0.215
No	1166	101	15.23	243	36	27.22	2.23 (1.72–2.89)	<0.0001	
Yes	260	23	15.00	122	26	38.53	2.93 (2.20–3.90)	<0.0001	
CVD history									0.448
No	1269	107	14.66	296	46	28.65	2.48 (1.99–3.09)	<0.0001	
Yes	157	17	19.57	69	16	40.83	2.10 (1.42–3.11)	0.0002	

Abbreviations: IR, incidence rate per 1000 person-years; Adj HR, IPW-adjusted hazard ratio; Int. *p*, interaction *p*-value; Prog, progression; Non-Prog, non-progression; CVD, cardiovascular disease. Reference group: non-progression within each subgroup. Adjusted for standardized IPW weights.

## Data Availability

The original contributions presented in this study are included in the article/Supplementary Material. Further inquiries can be directed to the corresponding authors.

## Supplementary Materials

The following supporting information can be downloaded at: https://www.mdpi.com/article/10.3390/jcm15124652/s1. Supplementary Table S1: Baseline Characteristics of Participants Included Versus Excluded From the IPW-Adjusted Cox Analysis (*n* = 1535 vs. *n* = 256). Supplementary Table S2: Sensitivity analysis using conventional multivariable Cox regression. Supplementary Table S3: Multiple-imputation sensitivity analysis for missing covariate data. Supplementary Table S4: Sensitivity Analysis Using an Alternative CAC Progression Threshold of ≥15 Agatston Units/Year. Supplementary Table S5: Distribution of Medication Use According to CAC Progression Status. Supplementary Table S6: Univariable and Multivariable Association Between Medication Use and CAC Progression. Supplementary Figure S1: Landmark cumulative-incidence curves for MACE according to CAC progression status. Supplementary Figure S2: Adjusted Hazard Ratios for MACE Across Joint Categories of CAC Progression and Statin Use.
