# Supplementary material for: Serial Coronary Artery Calcium Progression and Risk of Major Adverse Cardiovascular Events in an Asian Cohort
Source: J Clin Med. 2026 Jun 16;15(12):4652. doi: 10.3390/jcm15124652 (PMC13302396; doi:10.3390/jcm15124652)
Supplement: Supplementary file 1 [file jcm-15-04652-s001.zip › jcm-4339607-supplementary.pdf]

Supplementary Table S1. Baseline Characteristics of Participants Included Versus Excluded From the IPW–Adjusted Cox Analysis (*n* = 1,535 vs. *n* = 256)

Variable	Included (*n*=1,535)	Excluded (*n*=256)	*p*–value
Age, years, mean (SD)	53.9 (9.50)	53.5 (9.53)	0.496
Male sex, n (%)	1148 (74.8)	191 (74.6)	0.951
BMI, kg/m², mean (SD)	25.5 (3.71)	25.4 (3.46)	0.790
Diabetes mellitus, n (%)	155 (10.1)	11 (4.30)	0.003
Hypertension, n (%)	334 (21.8)	48 (18.8)	0.277
Hyperlipidemia, n (%)	356 (23.2)	35 (13.7)	0.0006
Cardiovascular disease, n (%)	187 (12.2)	39 (15.2)	0.173
SBP, mmHg, mean (SD)	127.5 (18.1)	128.5 (19.0)	0.629
DBP, mmHg, mean (SD)	78.6 (11.8)	80.0 (14.3)	0.383
hs–CRP, mg/L, mean (SD)	0.25 (0.69)	0.41 (0.87)	0.346
Homocysteine, µmol/L, mean (SD)	10.1 (3.30)	10.3 (NA)	0.963
BUN, mg/dL, mean (SD)	11.9 (4.06)	15.1 (8.56)	0.009
GFR, mL/min/1.73m², mean (SD)	90.9 (14.6)	92.0 (14.6)	0.307
Fasting glucose, mg/dL, mean (SD)	102.3 (26.0)	NA	NA
Total cholesterol, mg/dL, mean (SD)	202.8 (39.3)	184.9 (41.6)	0.038

Triglycerides, mg/dL, mean (SD)	153.7 (116.9)	169.4 (193.6)	0.723
HDL–C, mg/dL, mean (SD)	45.0 (12.5)	41.8 (10.1)	0.300
LDL–C, mg/dL, mean (SD)	124.9 (34.2)	107.8 (37.7)	0.023
Uric acid, mg/dL, mean (SD)	6.31 (1.48)	5.23 (1.25)	0.012
Antihypertensive, n (%)	11 (0.72)	5 (1.95)	0.066
Cardiac drug, n (%)	103 (6.71)	16 (6.25)	0.784
Diuretics, n (%)	40 (2.61)	2 (0.78)	0.076
Beta–blocker, n (%)	37 (2.41)	5 (1.95)	0.825
CCB, n (%)	131 (8.53)	7 (2.73)	0.001
RAS inhibitor, n (%)	96 (6.25)	3 (1.17)	0.0003
Antithrombotic, n (%)	144 (9.38)	15 (5.86)	0.067
Antidiabetic, n (%)	53 (3.45)	1 (0.39)	0.005
Statin, n (%)	140 (9.12)	4 (1.56)	<0.0001
CAC progression, n (%)	312 (20.3)	53 (20.7)	0.890
MACE events, n (%)	166 (10.8)	20 (7.81)	0.145

Abbreviations: BMI, body mass index; BUN, blood urea nitrogen; CAC, coronary artery calcium; CCB, calcium channel blocker; GFR, glomerular filtration rate; HDL–C, high–density lipoprotein cholesterol; hs–CRP, high–sensitivity C–reactive protein; LDL–C, low–density lipoprotein cholesterol; MACE, major adverse cardiovascular events; RAS, renin–angiotensin system; SBP, systolic blood pressure; SD, standard deviation.

Supplementary Table S2. Sensitivity analysis using conventional multivariable Cox regression

CAC progression category	HR	95% CI
Non–progression	Ref.	
Progression	1.85	1.29–2.64
20–49 Agatston units/year	1.44	0.88–2.34
≥50 Agatston units/year	2.44	1.54–3.88

Abbreviations: CAC, coronary artery calcium; CI, confidence interval; HR, hazard ratio.

Supplementary Table S3. Multiple–imputation sensitivity analysis for missing covariate data

CAC progression category	HR	95% CI
Non–progression	Ref.	
Progression	1.90	1.36–2.66
20–49 Agatston units/year	1.51	0.97–2.36
≥50 Agatston units/year	2.45	1.59–3.75

Abbreviations: CAC, coronary artery calcium; CI, confidence interval; HR, hazard ratio.

Supplementary Table S4. Sensitivity Analysis Using an Alternative CAC Progression Threshold of ≥15 Agatston Units/Year

CAC progression category	HR	95% CI
Non–progression	Ref.	
Progression	34.8	25.8–46.9
15–49 Agatston units/year	34.9	25.4–48.0
≥50 Agatston units/year	34.6	22.8–52.6

Abbreviations: CAC, coronary artery calcium; CI, confidence interval; HR, hazard ratio.

Supplementary Table S5. Distribution of Medication Use According to CAC Progression Status

Variable	Progression (n=365) n (%)	Non–Progression (n=1,426) *n* (%)	*p*–value
Antihypertensive	3 (0.82)	13 (0.91)	0.871
Cardiac drug	34 (9.32)	85 (5.96)	0.022
Diuretics	17 (4.66)	25 (1.75)	0.001
Beta–blocker	11 (3.01)	31 (2.17)	0.344
CCB	39 (10.7)	99 (6.94)	0.017
RAS inhibitor	35 (9.59)	64 (4.49)	0.0001
Antithrombotic	55 (15.1)	104 (7.29)	<0.0001
Antidiabetic	23 (6.30)	31 (2.17)	<0.0001
Statin	49 (13.4)	95 (6.66)	<0.0001

Abbreviations: CCB, calcium channel blocker; RAS, renin–angiotensin system.

Supplementary Table S6 Univariable and Multivariable Association Between Medication Use and CAC Progression

Variable	Crude OR	95% CI	p–value	Adjusted OR	95% CI	*p*–value
Cardiac drug	1.62	(1.07 – 2.46)	0.023	0.18	(0.02 – 2.23)	0.183
Diuretics	2.74	(1.46 – 5.13)	0.002	2.36	(0.17 – 33.5)	0.527
CCB	1.60	(1.09 – 2.37)	0.018	0.69	(0.14 – 3.46)	0.647
RAS inhibitor	2.26	(1.47 – 3.47)	0.0002	0.67	(0.09 – 4.80)	0.688
Antithrombotic	2.26	(1.59 – 3.20)	<0.0001	2.12	(0.24 – 18.5)	0.496
Antidiabetic	3.03	(1.74 – 5.26)	<0.0001	5.82	(0.56 – 60.7)	0.140
Statin	2.17	(1.51 – 3.13)	<0.0001	2.03	(0.30 – 13.5)	0.466

Abbreviations: CCB, calcium channel blocker; CI, confidence interval; OR, odds ratio; RAS, renin–angiotensin system.

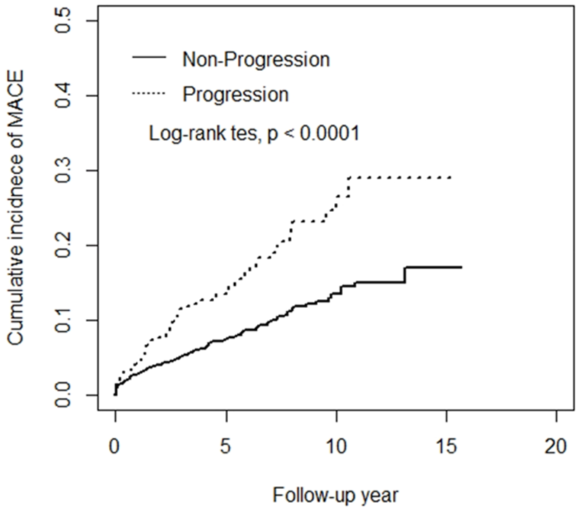

Figure S1. Landmark cumulative–incidence curves for MACE according to CAC progression status.

Follow–up began at the second CAC scan. CAC progression was defined as an annualized Agatston score increase ≥20 units/year. The cumulative incidence of MACE was higher among participants with CAC progression than among non–progressors (log–rank test, $p < 0.0001$).

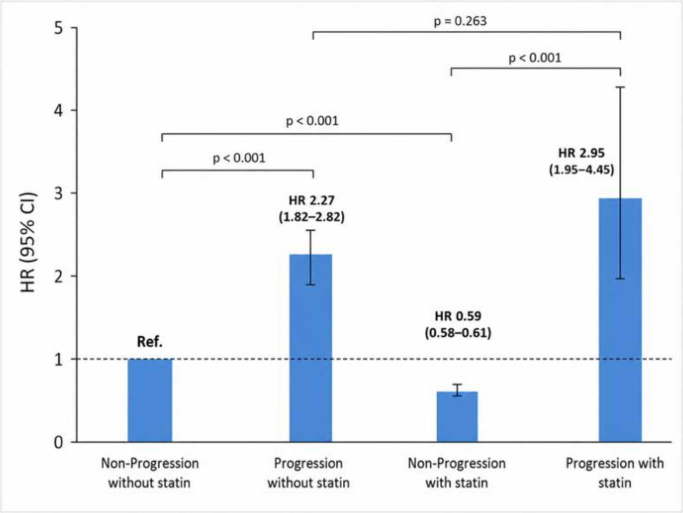

Supplementary Figure S2. Adjusted Hazard Ratios for MACE Across Joint Categories of CAC Progression and Statin Use.

IPW–adjusted hazard ratios for MACE across four groups defined by statin use and CAC progression status, with the non–progression without statin group serving as the reference. The p–values shown above the brackets indicate pairwise comparisons between selected joint categories. The CAC progression × statin interaction was assessed separately from these pairwise comparisons by including an interaction term in the landmark IPW–adjusted Cox model; the interaction p–value is reported in the Results section. Error bars indicate 95% confidence intervals.
